# A graphene-based hybrid material with quantum bits prepared by the double Langmuir–Schaefer method†

**DOI:** 10.1039/c9ra04537f

**Published:** 2019-08-02

**Authors:** Jakub Hrubý, Vinicius T. Santana, Dmytro Kostiuk, Martin Bouček, Samuel Lenz, Michal Kern, Peter Šiffalovič, Joris van Slageren, Petr Neugebauer

**Affiliations:** Central European Institute of Technology, CEITEC BUT Purkyňova 656/123 61200 Brno Czech Republic petr.neugebauer@ceitec.vutbr.cz; Institute of Physics, Slovak Academy of Sciences Dúbravská cesta 9 84511 Bratislava Slovakia; Institute of Physical Engineering, Faculty of Mechanical Engineering, Brno University of Technology Technická 2 61669 Brno Czech Republic; Institute of Physical Chemistry, University of Stuttgart Pfaffenwaldring 55 70569 Stuttgart Germany

## Abstract

The scalability and stability of molecular qubits deposited on surfaces is a crucial step for incorporating them into upcoming electronic devices. Herein, we report on the preparation and characterisation of a molecular quantum bit, copper(ii)dibenzoylmethane [Cu(dbm)_2_], deposited by a modified Langmuir–Schaefer (LS) technique onto a graphene-based substrate. A double LS deposition was used for the preparation of a few-layer-graphene (FLG) on a Si/SiO_2_ substrate with subsequent deposition of the molecules. Magnetic properties were probed by high-frequency electron spin resonance (HF-ESR) spectroscopy and found maintained after deposition. Additional spectroscopic and imaging techniques, such as Raman spectroscopy (RS), X-ray photoelectron spectroscopy (XPS), atomic force microscopy (AFM), and scanning electron microscopy (SEM) were performed to characterise the deposited sample. Our approach demonstrated the possibility to utilise a controlled wet-chemistry protocol to prepare an array of potential quantum bits on a disordered graphene-based substrate. The deployed spectroscopic techniques showed unambiguously the robustness of our studied system with a potential to fabricate large-scale, intact, and stable quantum bits.

## Abstract 2

Graphene-based hybrid material with array of copper(ii)-based quantum bits was prepared by a wet-chemistry protocol and characterised by HF-ESR, XPS, Raman, and AFM.

## Graphical abstract

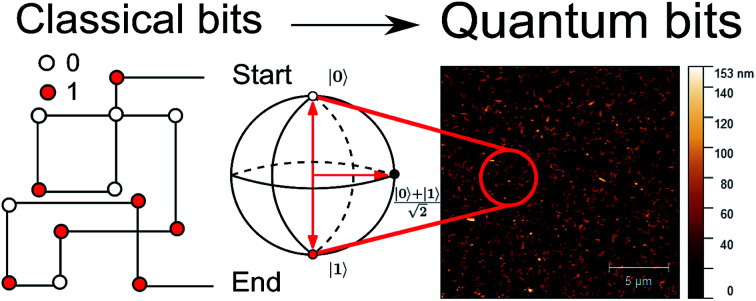

## 1 Introduction

Core computational units based on molecular architecture present one way in pursuing a quantum computer, a device which could substantially change all fields of human activity from complex structural biology to finance.^1,2^ The core is based on the principle of quantum superposition in which multiple states can be accessed at the same time; in contrast to classical zeroes and ones, which are used in current linear computational technology. A quantum computer could outperform any classical one in factoring numbers and searching a database.^3^ In the search for novel approaches to standard silicon-based electronics, several routes were proposed. One of the promising routes is the merge of electronics controlled by spin of an electron (spintronics)^4^ and molecular electronics.^5^

Nowadays, there are several approaches in research of quantum bits (qubits) aiming at controlling spin states which is the origin of magnetism in a matter. Crystal-structure defects with a net spin such as nitrogen vacancies in diamond^6^ or double vacancies in silicon carbide^7–9^ are currently under investigation due to their potential for quantum information processing. However, the issue related to the usability of such centres lies in the production of defects as they can be prepared either by electron or ion bombardment, both of which create a random distribution of defects in the material.^10^ These solid-state systems of defect sites prepared by lithographic means offer only a limited synthetic control over their electronic and magnetic properties, which is crucial in integration with large scale devices. Another approach relies on using electron spins provided within magnetic molecules with unpaired electrons. The advantages over solid-state systems are mainly the reproducible fabrication, the ability to fine-tune magnetic properties by changing the ligand environment of an active-metal site, and the possibility to form large-scale ordered arrays. Hence, molecular magnets are also promising candidates for qubits as they offer unpaired electrons in stable molecular environment.^11^

Herein, we have decided to challenge two out of five DiVincenzo's criteria postulated on the brink of quantum computer development era in 2000.^12^ We addressed namely characterisation and scalability of the copper(ii)dibenzoylmethane [Cu(dbm)_2_], where dbm stands for dibenzoylmethane, with linear chemical formula: C_30_H_22_CuO_4_, which is a transition-metal complex with copper as a central atom surrounded by two dbm ligands. It is a potential qubit with coherence time of dozens of microseconds at low temperatures,^13^ which is a time usable for quantum information processing. To circumvent abovementioned challenges with a host material for spins, we have opted for a few-layer-graphene (FLG) which is a stacked single graphite sheet known as graphene^14^ famous for its interesting properties such as high electron mobility, mechanical strength and thermal conductivity.^15–18^ The FLG in our sample provides a conductive substrate for qubits with a possibility to apply a gate voltage in order to have a control over spin states. This lead us to a production of a graphene-based hybrid material *via* controlled wet-chemistry protocol with an intact and robust molecular qubit.

Currently, there are several methods of how to prepare graphene, by original micro-mechanical cleavage,^19^ chemical vapour deposition (CVD),^20^ epitaxial growth from SiC,^21^ or liquid-phase exfoliation.^22^ The last method is possibly the best option for scalable production of graphene-based hybrid materials, thin conductive films, and thermal pastes.^23,24^ In order to obtain exfoliated graphene, there is a need for applying external force to graphite flakes, such as ultrasonic vibrations.^25^ Recently, there has been a growing interest in graphene-based materials that led to the chemically engineered graphene-based magnetic,^26^ opto-electrical,^27,28^ and bio-sensing devices.^29–31^ Besides, the supramolecular approach to graphene resulting in physisorption of magnetic materials directly onto the substrate *via* π–π stacking without any further functionalisation appears to be feasible.^32^ The ability to control the magnetic properties of graphene-based hybrid materials is crucial for the further development of carbon-based devices.^33,34^

We have prepared a hybrid structure of a deposited quantum bit represented by organometallic complex [Cu(dbm)_2_] onto FLG-covered Si/SiO_2_ substrate and investigated its stability and robustness by Raman spectroscopy, XPS, and HF-ESR. Our proposed wet-chemistry-based approach can be considered as a complementary study to recently published one in which [Cu(dbm)_2_] was successfully evaporated onto Au(110) substrate by organic molecular beam deposition,^35^ and thus showing both deposition routes are viable for this qubit.

## 2 Experimental section

### 2.1 Synthesis of [Cu(dbm)_2_]

The synthesis of [Cu(dbm)_2_] complex followed previously described procedure.^36^ Copper(ii) chloride dihydrate (1 mmol, 170 mg) was dissolved in water (15 mL). Simultaneously, a solution of dibenzoylmethane (2 mmol, 458 mg) and potassium hydroxide (1.4 mmol, 80 mg) in ethanol/water mixture with 15 : 1 ratio was added drop-wise to the copper solution. The resultant solution was stirred by a magnetic stirrer for 30 min. Finally, the precipitate was separated by a paper filtration and washed once by pure ethanol and demineralised water with consecutive vacuum filtration for 12 h. The reaction yield of the synthesis was fair (347 mg, 66%). Elemental analysis performed on [Cu(dbm)_2_]; anal. calc. for C_30_H_22_CuO_4_, *M*_w_ = 510.04: C – 70.65; H – 4.35. Found: C – 71.73; H – 4.58.

### 2.2 Langmuir–Schaefer (LS) deposition

The expanded and milled graphite (SGL-carbon) was dispersed at the concentration of 10 mg mL^−1^ in *N*-methyl-2-pyrrolidone (NMP) and was sonicated for 60 min at room temperature in an ultrasonic bath. Since the graphene layers tend to re-aggregate due to the weak van der Waals forces acting between them, the stabilisation agent NMP was used as a sonication medium. It prevented graphene layers from re-aggregation by minimising the interfacial tension between the graphene sheets and the medium.^37^ The prepared FLG suspension in NMP was centrifuged in order to separate supernatant from sediment. The suspension was centrifuged at 10 320 rpm for 30 min. Afterwards, the supernatant was taken and left further to settle in a fridge (*T* = 2–3 °C) for a month. The final concentration of graphene in the suspension was 0.1–0.2 mg mL^−1^ with the average lateral size of nano-sheets: 300 nm and the average thickness of nano-sheets from 5–10 nm (*i.e.* 10–20 layers). Details of FLG characterisation are in Fig. S1 in the ESI.†

The first deposition took place in the first Langmuir–Blodgett trough (KSV NIMA), where we deposited FLG onto a boron-doped Si(100) p-type substrate with a 500 nm thick layer of SiO_2_ (see Fig. S2 in the ESI†) and resistivity *ρ* = 10–20 Ω cm. The 10 mL of FLG dispersion in NMP was gently spread onto the water subphase using a microlitre syringe. The pH of the water subphase was lowered to 4 by the addition of HCl, which is known to improve the floatation of nanoscale materials. The FLG Langmuir film compressed at the surface pressure of 38 mN m^−1^ was transferred onto the Si substrates by the controlled removal of the water subphase. Prior to deposition, the Si samples were submersed into the water subphase, having a tilt of approximately 5° with respect to the air/water interface. During the water removal, the tilt of the samples facilitated an increased spatial homogeneity of the deposited FLG layers due to a predefined sliding direction of the three-phase boundary. After the first FLG deposition was made, samples were annealed at 800 °C for 30 min. This step helped to remove the remaining water from the deposition and to stabilise layer on the substrate.

**Fig. 1:**
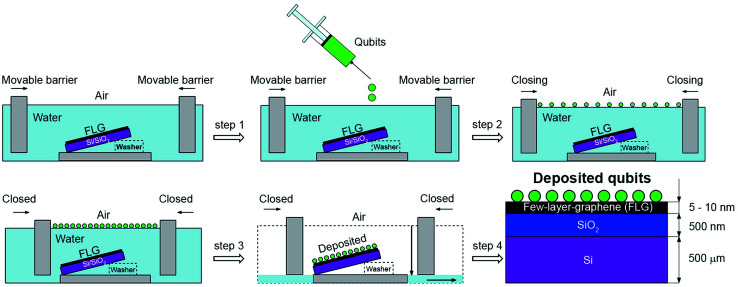
Scheme of modified Langmuir–Schaefer deposition process. It started by Langmuir–Blodgett trough filled with deionised water sub-phase. Step 1: the solution of [Cu(dbm)_2_] in chloroform was injected onto a water subphase. The [Cu(dbm)_2_] formed an oil-like structure on the water/air interface. Step 2: the movable barriers closing until the molecules formed a tight layer. Step 3: the movable barriers were fully closed and water subphase was carefully pumped out under the movable barrier until the water level reached the substrate and the layer was deposited. Step 4: the final layer was composed of [Cu(dbm)_2_] qubits deposited onto the FLG-covered substrate.

Fig. 1 depicts the scheme for the second LS deposition where we used second (see Fig. S4 in the ESI†) Langmuir–Blodgett trough for the qubit deposition (KSV NIMA) filled with deionised water (PURELAB Classic ELGA). A 5 mM solution of [Cu(dbm)_2_] in chloroform (Alfa Aesar, 99.8%) was dropped (*V* = 1.4 mL) onto a water sub-phase until no further spreading was visible. Compounds formed an oil-like structure on the water/air interface. The movable barriers were closed to a certain surface pressure estimated from blank measurements (see Fig. S5 in the ESI†). The main advantage of this method is in the possibility to use manifold of various substrates at the same time during the deposition, limited only by the dimension of the trough. The final multi-layered material was composed of [Cu(dbm)_2_] deposited onto the FLG-covered substrate (see Fig. S6 in the ESI†).

### 2.3 Raman spectroscopy (RS)

Raman spectra of the reference [Cu(dbm)_2_] powder were acquired on confocal Raman microscope WITec Alpha300 R+. All other spectra were acquired on confocal Raman microscope Renishaw inVia. Both instruments were equipped with the excitation laser source with 532 nm wavelength.

### 2.4 X-ray photoelectron spectroscopy (XPS)

X-ray photoelectron (XPS) measurements were carried out with a Kratos Axis Supra spectrometer at room temperature and ultra-high vacuum (UHV) conditions. The instrument was equipped with monochromatic Al Kα source 1486.6 eV (15 mA, 15 kV), and hemispherical analyser with hybrid magnetic and electrostatic lens for enhanced electron collection. Survey and detailed XPS spectra were acquired at normal emission with the fixed pass energy of 160 eV and 40 eV, respectively. Slot aperture 700 × 300 μm^2^ was used during the acquisition. All spectra were charge-corrected to the hydrocarbon peak set to 284.8 eV. The Kratos charge neutralizer system was used on all specimens. The inelastic backgrounds in all spectra were subtracted according to Shirley method.^38^ Data analysis was based on a standard deconvolution method using mixed Gaussian (G) and Lorentzian (L) line shape (G = 70% and L = 30%, Gaussian–Lorentzian product) for each component in the spectra. Spectra were analyzed using CasaXPS software (version 2.3.18).

### 2.5 Electron spin resonance (ESR)

High-frequency/-magnetic field ESR spectra (HF-ESR) were acquired on a home-built spectrometer featuring a VDI signal generator, a VDI amplifier-multiplier chain, a quasi-optical bridge (Thomas Keating), an Oxford Instruments 15/17 T solenoid cryomagnet and a QMC Instruments InSb hot-electron bolometer.^39^ The reference powder sample of the [Cu(dbm)_2_] was studied as a pressed polytetrafluoroethylene-wrapped powder pellet and the final sandwich-like deposited hybrid material as *ø* 5 mm sample (see Fig. S7 in ESI† for more information). All ESR spectra were simulated using the EasySpin toolbox for Matlab.^40^

## 3 Results and discussion

**Fig. 2:**
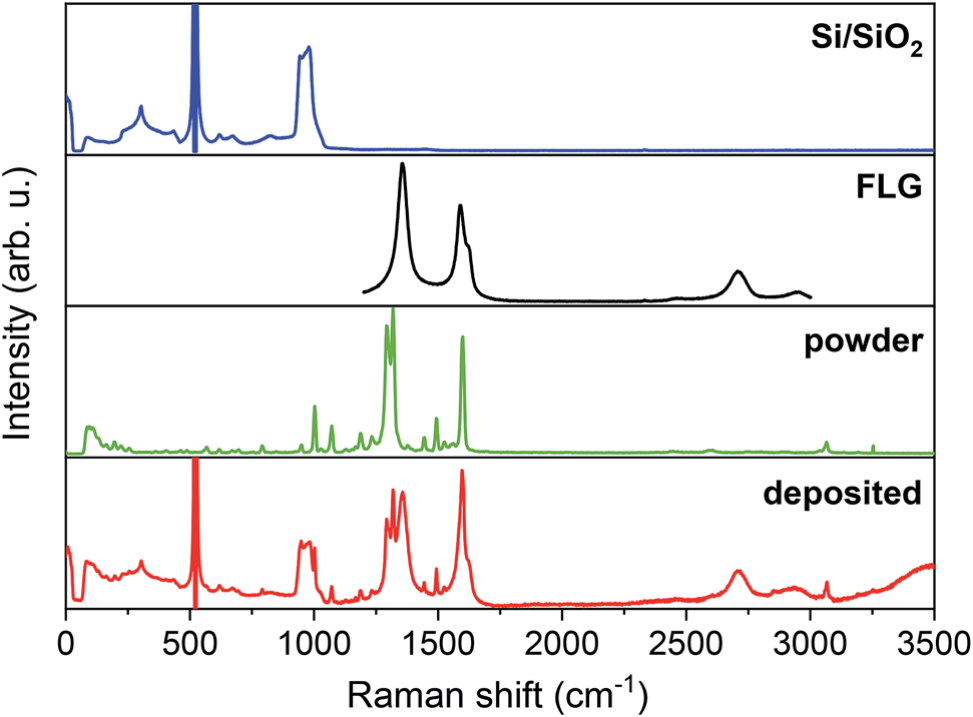
Comparison of Raman spectra, from top to bottom, of Si/SiO_2_ substrate, FLG region, powder [Cu(dbm)_2_] and deposited [Cu(dbm)_2_].

We performed Raman spectroscopy to explore vibrational modes of individual components in the resultant hybrid material and made a comparison in order to confirm the intactness of our studied system. Fig. 2 shows Raman spectra from top to bottom of a bare Si/SiO_2_ substrate, FLG, [Cu(dbm)_2_] powder, and the final deposited sample.

For the main comparison purpose the evident 2 transverse acoustic (TA) phonon band at 304 cm^−1^, the most intense main silicon transverse optic (TO) phonon band at 521 cm^−1^, and 2 transverse optic phonon band spanning from 943 cm^−1^ to 983 cm^−1^ were observed for the Si/SiO_2_ substrate.^41,42^ In the case of the FLG, six distinct peaks were found. The D band at 1356 cm^−1^, G band at 1588 cm^−1^, D′ band at 1621 cm^−1^, G′ band at 2709 cm^−1^, G + D band at 2953 cm^−1^, and in multi-layered material 2D′ peak which can be attributed to the second order of the intra-valley D′ for graphene layers.^43^ In the case of FLG, the disorder-induced D band, which is approximately at half of the frequency of G′ band, is the most intense peak signalling for a largely disordered graphitic substrate. The G band corresponds to doubly degenerate (iTO and LO) phonon mode at the Brillouin zone centre. The G band is the only band coming from a normal first-order Raman scattering process in graphene. On the contrary, the G′ and D bands originate from a second-order process involving two iTO phonons near the *K* point of the first Brillouin zone for the G′ band or one iTO phonon and one defect in the case of the D band.^44^ All peaks were present both in the bare FLG on Si/SiO_2_ substrate and the final deposited sample. Peak positions found for [Cu(dbm)_2_] were in accordance with the previous Raman, infrared and in-depth theoretical studies performed on this system and dbm ligand.^45,46^ The intensity of peaks is denoted from very strong to very weak and peaks assignment is provided based on the previous theoretical and experimental study^45^ (see Table S8 in the ESI†). Raman spectra confirmed unequivocally all vibrational peaks for [Cu(dbm)_2_] to be present for both bulk as well as deposited molecules.

**Fig. 3:**
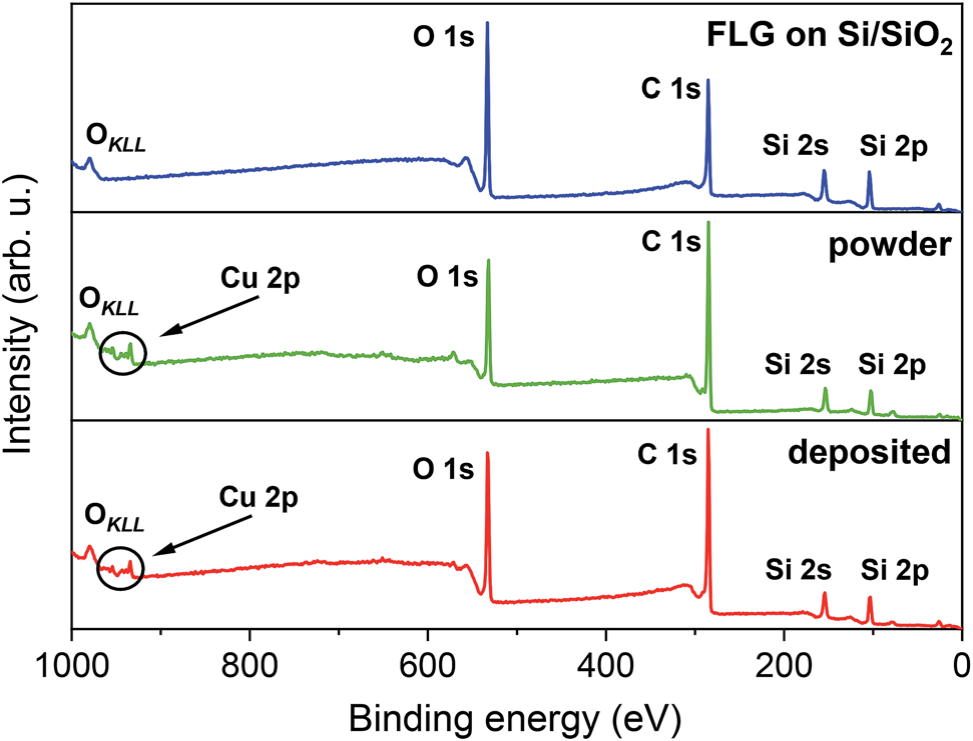
XPS survey spectra comparison, from top to bottom, of FLG on Si/SiO_2_ substrate, powder [Cu(dbm)_2_] and deposited [Cu(dbm)_2_].

The chemical composition of FLG on Si/SiO_2_, [Cu(dbm)_2_] powder, and [Cu(dbm)_2_] on FLG was probed by means of XPS. Fig. 3 shows the survey spectra that exhibit O 1s, C 1s, Si 2s, and Si 2p photoelectron peaks and a visible O_KLL_ Auger peak.

Silicon peaks in the [Cu(dbm)_2_] powder spectra are present due to the silicon adhesive used on polyimide-based double-sided tape which hosted as-synthesised [Cu(dbm)_2_] powder. Highly resolved spectra of C 1s, O 1s, and Cu 2p revealed chemical bond type. Slight difference in binding energies for corresponding peaks could be attributed to changed variations in the surrounding chemical environment.^47^ The best-fit parameters were found by applying physical constraints to components forming the overall spectrum based on stoichiometry, bond strength, and electronegativity.^47,48^ However, due to the ambient conditions during the LS deposition technique and carbon-based FLG background we were not able to quantify peak intensities and relate them with molecular structure.

**Fig. 4:**
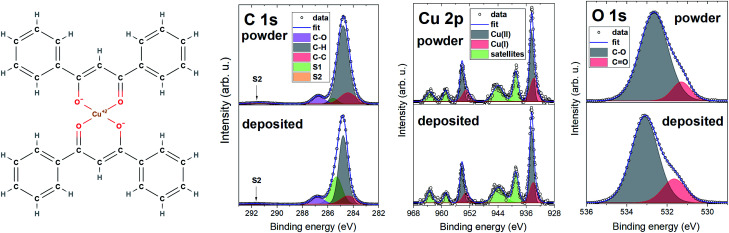
Molecular structure of [Cu(dbm)_2_] with XPS comparison of powder and deposited sample with detailed regions for C 1s, Cu 2p, and O 1s.

Fig. 4 shows the chemical structure of [Cu(dbm)_2_] and detailed XPS spectra characterised by main peaks at 284.8 eV, 532.7 eV, and 934.5 eV for C 1s, O 1s, and Cu 2p_3/2_ core levels, respectively. The C 1s and O 1s experience a slight shift, towards lower binding energy in the deposited spectra. This can be caused by image-charge screening effect.^49^ The chemical structure suggests expecting components in C 1s from both carbon atoms forming aromatic rings (C–C, C–H) and carbon atoms bound to an oxygen atom (C=O, C–O). Photoelectrons from aromatic rings appear at lower binding energy, whereas carbon atoms bound to oxygen create a strong shoulder feature at 286.8 eV. We have also detected two satellite features S1 and S2, which are typical in photoemission of organic molecules due to relaxation processes resulting in the creation of a core-hole.^50^ The increase in the intensity of S1 satellite at the expense of C–H in the deposited spectrum can be attributed to the physisorption on FLG substrate, image-charge screening effect and charge neutralization processes.

The O 1s core level line showed, beside the main component at 532.7 eV for powder and 533.1 eV for deposited, a shoulder component at lower binding energy which was identified as C=O bond. The Cu 2p photoemission line features two main peaks Cu 2p_3/2_ and Cu 2p_1/2_ at 934.5 eV and 954.3 eV, respectively, separated by 19.8 eV. We have not evidenced any measurable energy shift in binding energy of Cu 2p peaks in powder and deposited spectra. Both powder and deposited peaks exhibit a complex satellite structure, which serves as a fingerprint of having Cu atoms with a 2+ oxidation state.^51–53^ This corroborates the preserved oxidation state of Cu(ii) after the LS deposition process. Strong shake-up satellites visible in regions (937–947) eV and (957–967) eV stem from electronic relaxation effects caused by electron-correlation effects in the open-shell D bands.^51^ We have also evidenced a presence of Cu(i) component, shifted towards lower binding energy in comparison with Cu(ii). This is a common reduction process for Cu(ii)-based systems even without apparent reducing agent.^54^ Studied sample was exposed to air, where Cu_2_O is the final product of air exposure,^55^ and was in contact with excessive amount of water during the LS deposition. Cu(ii) can also be partially reduced to Cu(i) by excess free electrons in FLG layer directly in contact with [Cu(dbm)_2_]. XPS results are matching the findings recently gained on this system thermally evaporated on Au(110).^35^

**Fig. 5:**
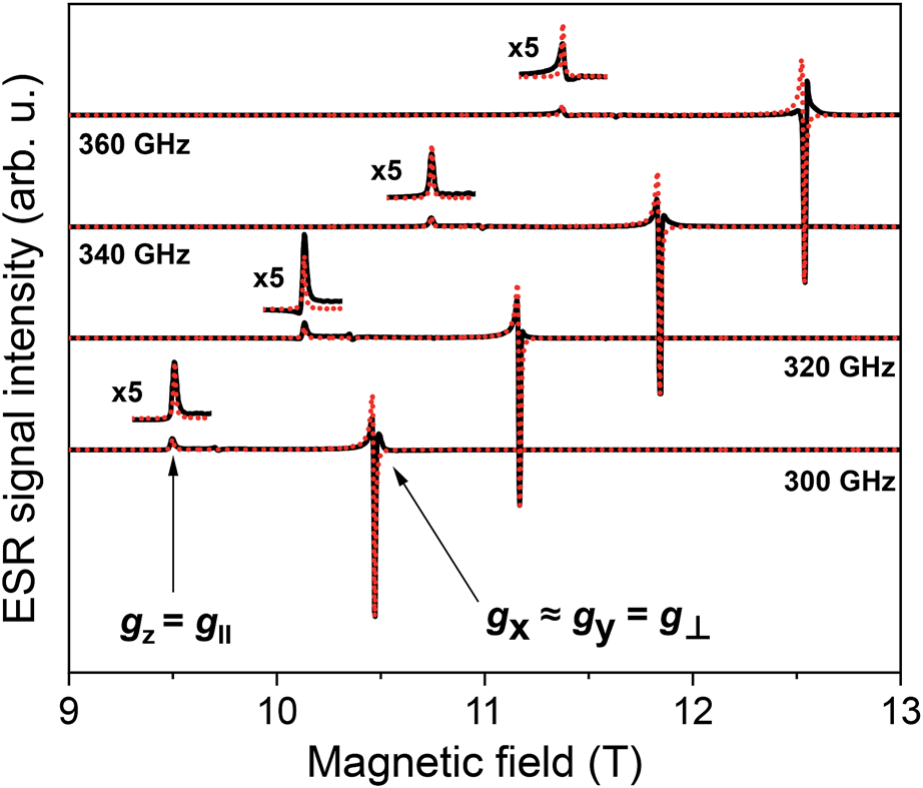
HF-ESR powder spectra of [Cu(dbm)_2_] for four different frequencies, 300 GHz, 320 GHz, 340 GHz, and 360 GHz, respectively, from 9 T to 13 T measured at 5 K.

We have also investigated the intrinsic magnetic properties of [Cu(dbm)_2_] before and after deposition by means of multi-frequency HF-ESR spectroscopy. Fig. 5 shows HF-ESR spectra for powder [Cu(dbm)_2_] from which we extracted basic spin Hamiltonian parameters such as *g*-factor originating from one unpaired electron of Cu(ii) ion in 3d^9^ electron configuration. Eqn (1) describes the used spin Hamiltonian constructed by taking into account Zeeman contribution:

1 *Ĥ* = *Ĥ*_Zeeman_ = *μ*_B_*B⃑*^T^*g̃S⃑*,

where *μ*_B_ is Bohr magneton, *h* is Planck constant, *B⃑*^T^ is magnetic field, *g̃* is *g*-factor tensor, *S⃑* is total spin of system, in this case *S* = 1/2. All powder spectra were fitted with the spin Hamiltonian parameters as follows: *g*_*x*_ = 2.0480, *g*_*y*_ = 2.0470, *g*_*z*_ = 2.2570. Parameters used in the fitting procedure are in fair agreement with previously published results.^13,35,56^ The slight misalignment and intensity decrease in spectra can be attributed to calibration of magnetic field and lock-in phase changes during the measurements. The difference between frequencies can also be assigned to different power distribution profile from a microwave source.

**Fig. 6:**
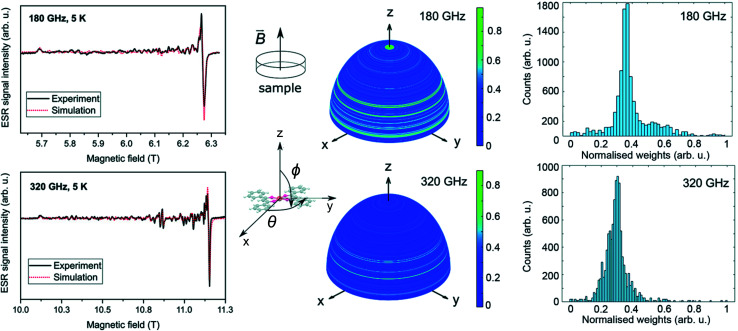
Left: HF-ESR spectra of deposited [Cu(dbm)_2_] for two different frequencies of 180 GHz and 320 GHz measured at 5 K. Middle: visualisation of integration sphere with 10 000 equally spaced points illustrating distribution of resonance lines. In our experimental setup the external magnetic field *B⃑* was perpendicular to the surface plane of the sample. The colour scale bar corresponds to the relative weight distribution for the simulated spectra in every direction. The angles 0 < *θ* < 2π and 0 < *ϕ* < π denote the Euler angles. The simulated spectra were expectably sensitive mostly to changing *ϕ*. Right: histogram of assigned weight distribution from an integration sphere, note that 320 GHz is more detailed than 180 GHz, demonstrating that by using higher frequencies, it is possible to get more resolved and finer distribution.

Fig. 6 shows the HF-ESR spectra of deposited [Cu(dbm)_2_] onto FLG-covered substrate with additional statistical analysis of resonance lines distribution. We observed a slight difference in *g*_*x*_ and *g*_*y*_ compared to bulk powder measurements. Both deposited spectra were fitted with the spin Hamiltonian parameters as follows: *g*_*x*_ = 2.0503, *g*_*y*_ = 2.0499, *g*_*z*_ = 2.2590. This shift can be caused by stacking of physisorbed [Cu(dbm)_2_] on FLG-based substrate.

We observed many absorption lines of a small intensity distributed between magnetic field values that correspond to resonances parallel and perpendicular to the plane of the molecules (*i.e.* between *B*∥*z* and *B*∥*xy* plane) for both frequencies 180 GHz and 320 GHz. We statistically evaluated these additional peaks in both spectra by simulating the ESR absorption equivalent to individual micro-crystallites in the sample. We have rotated the *g̃* tensor obtained from the bulk powder samples and performed a simulation over a sphere with 10 000 equally spaced points generated by the Repulsion algorithm,^57^ attributing a weight to the intensity of the spectrum in each direction. The weight is equivalent to the amount of molecules at a particular orientation while the counts in the histogram in Fig. 6 corresponds to the total number of orientations with a given weight. This result shows that there is not an effective selective orientation of the micro-crystallites formed on the surface, even though about 5% of them are distributed around the green regions in the integration hemispheres. The other 95% is randomly distributed when taking into account the roughness of FLG surface. The effect of the distribution of micro-crystallites is less pronounced at 180 GHz because the magnetic field resolution *δB* between adjacent absorption lines is smaller at lower frequencies (*B* = *hν*/*gμ*_B_). A simulation made by changing solely the frequency parameter shows that the double intensity at the same directions produces broader and smaller peak-to-peak intensities in the same regions. The pattern on integration hemispheres appears similar for both 180 GHz and 320 GHz spectra, however, the histogram for 320 GHz is more detailed. This can be assigned to an inherent property of going into higher frequencies in ESR in similar cases.

This multi-resonance line effect was also previously observed and discussed for the imperfectly dissolved frozen-solution of [Cu(dbm)_2_] in chloroform and called as a “single-crystal effect”.^58,59^ In our case on a surface, these lines are assigned to an incomplete distribution of the orientations of micro-crystallites.

## 4 Conclusions

To conclude, we have deposited and characterised molecular quantum bit based on Cu(ii) ion on a surface. We have demonstrated a possibility to produce large arrays of graphene-based hybrid materials with quantum bits by using a double Langmuir–Schaefer deposition. We have taken advantage of using disordered graphene substrate as a template matrix for physisorption of [Cu(dbm)_2_] which we have further characterised mainly to pinpoint the intactness and robustness of our system. The biggest advantage of our wet-chemistry based deposition protocol is the scalability and usability of whichever substrate with size limitations only by the dimensions of a LS trough. On the contrary, the access of atmospheric moisture and oxygen in the form of water can lead to abrupt decomposition with a limited amount of systems withstanding the air/water interface and a mixture of solvents during deposition, and thus is not generally applicable to every compound. To the best of our knowledge, there is no established database or a rule helping to determine properties of quantum bits on surfaces, and thus every system has to be treated individually. In this regard, there is a space for improving the deposition conditions by using inert nitrogen or argon atmosphere, water purification, as well as to fine-tune deposition parameters in molecular concentration and volume used. Additionally, the potential to use specially tailored ligands surrounding the active Cu(ii) ion offers possibilities of adjusting the adhering properties as well as hydrophobicity. Our work demonstrated a scalable deposition of quantum bits onto graphene-based substrate providing promising prospects of a wet-chemistry-based preparation route. Therefore, these molecular qubits in connection with conductive substrate seem to be the potent route in pursuing a quantum computation. Moreover, we have demonstrated that HF-ESR combined with statistical approach can be a useful tool for description of molecular distribution based on magnetic anisotropy on surfaces.

## Conflicts of interest

There are no conflicts to declare.

## Supplementary Material

RA-009-C9RA04537F-s001
