# Supplementary material for: A graphene-based hybrid material with quantum bits prepared by the double Langmuir–Schaefer method
Source: RSC Adv. 2019 Aug 2;9(42):24066–73. doi: 10.1039/c9ra04537f (PMC9069494; doi:10.1039/c9ra04537f)
Supplement: RA-009-C9RA04537F-s001 [file RA-009-C9RA04537F-s001.pdf]

Electronic Supplementary Material (ESI) for RSC Advances.
This journal is © The Royal Society of Chemistry 2019

ELECTRONIC SUPPORTING INFORMATION

Graphene-based hybrid material with quantum bits prepared by double Langmuir–Schaefer method

Jakub Hrubý,[a] Vinicius T. Santana,[a] Dmytro Kostiuk,[b] Martin Bouček,[c] Samuel Lenz,[d] Michal Kern,[d] Peter Šiffalovič,[b] Joris van Slageren[d] and Petr Neugebauer[a*]*

[a]Central European Institute of Technology, CEITEC BUT, Purkyňova 656/123, 61200 Brno, Czech Republic

[b]Institute of Physics, Slovak Academy of Sciences, Dúbravská cesta 9, 84511 Bratislava, Slovakia

[c]Institute of Physical Engineering, Faculty of Mechanical Engineering, Brno University of Technology, Technická 2, 61669 Brno, Czech Republic

[d]Institute of Physical Chemistry, University of Stuttgart, Pfaffenwaldring 55, 70569 Stuttgart, Germany

This Supporting Information contains:

1) Characterisation of a few-layer-graphene (FLG) by optical microscopy, AFM, SEM, and HR-TEM.
2) Langmuir – Schaefer deposition setup scheme.
3) Isotherms obtained during blank deposition of [Cu(dbm)$_2$] without substrates and the actual deposition.
4) SEM and AFM characterisation of deposited [Cu(dbm)$_2$] on FLG
5) High-frequency ESR setup scheme.
6) Raman comparison of peaks found with their assignment.

1) Characterisation of a few-layer-graphene (FLG) by optical microscopy, AFM, SEM, and HR-TEM.

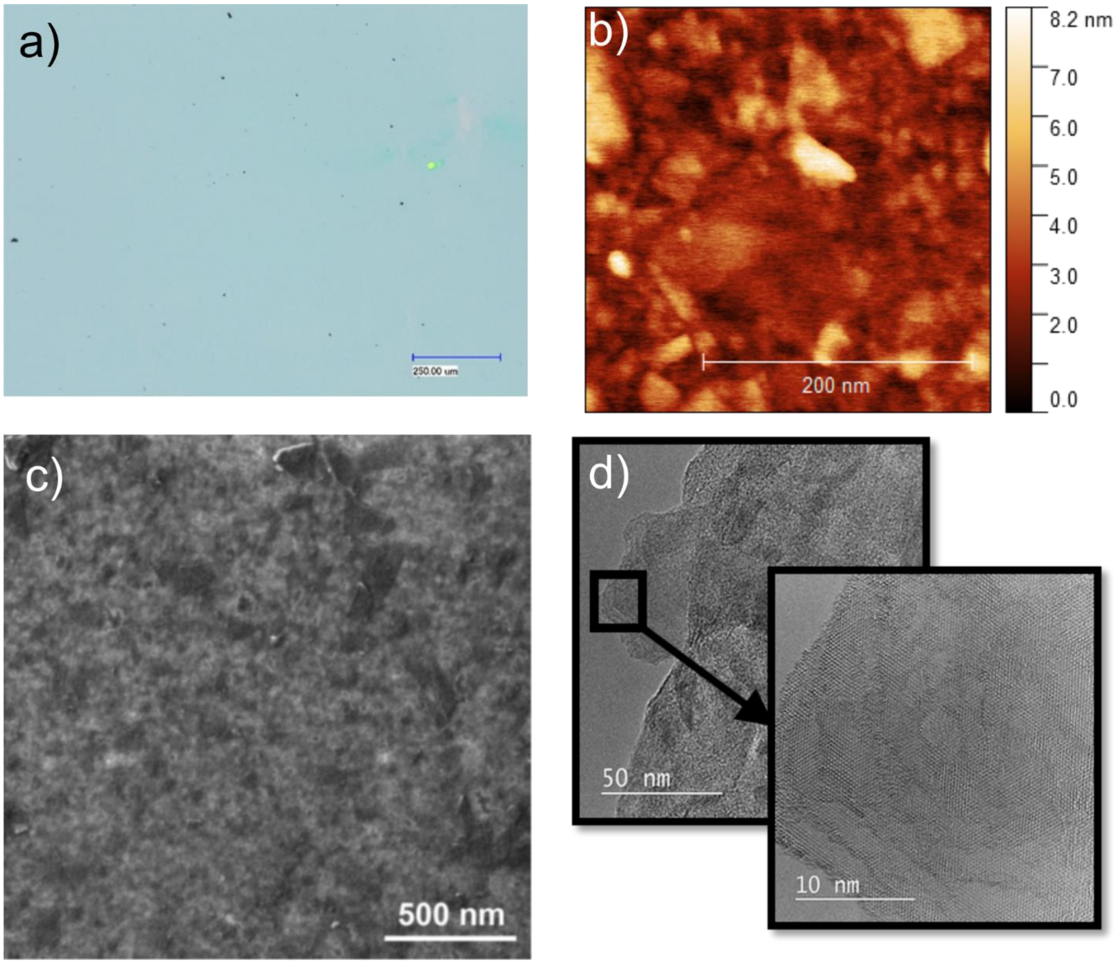

Figure S1. Characterisation of FLG layer for its thickness and uniformity by a) optical microscopy, b) AFM image, c) SEM image, and d) HR-TEM image.

Additionally, the four-point-probe resistance measurements were performed on bare Si/SiO_2, FLG, and also a CVD graphene sample and revealed 10^6 times higher conductivity of FLG compared to Si/SiO_2 and around 50 times higher conductivity for CVD graphene compared to FLG. Therefore, it is possible to tune conductivity based on the used substrate.[1]

2) Langmuir - Schaefer deposition setup scheme.

Figure S2 shows experimental setup for modified Langmuir–Schaefer deposition which started by injecting the FLG suspension onto a subphase (deionised water in this case) surface (1). The movable barriers (2) were slowly closed and reduced the surface area of a trough top (1). The process of a layer formation was observed by an optical microscope (3) with a visual output (4) to a monitor, and a Wilhelmy plate* made out of paper for monitoring the surface pressure (5). The (6) is the Si/SiO_2 substrate on which the FLG deposition took place. Finally, the suction pump (7), by which the water was pumped out, and thus the water level was lowered and the deposition was done. The term modified stands for the deposition carried out by elevating and tilting the substrate. This was done by placing metal nuts of different sizes underneath the substrates.

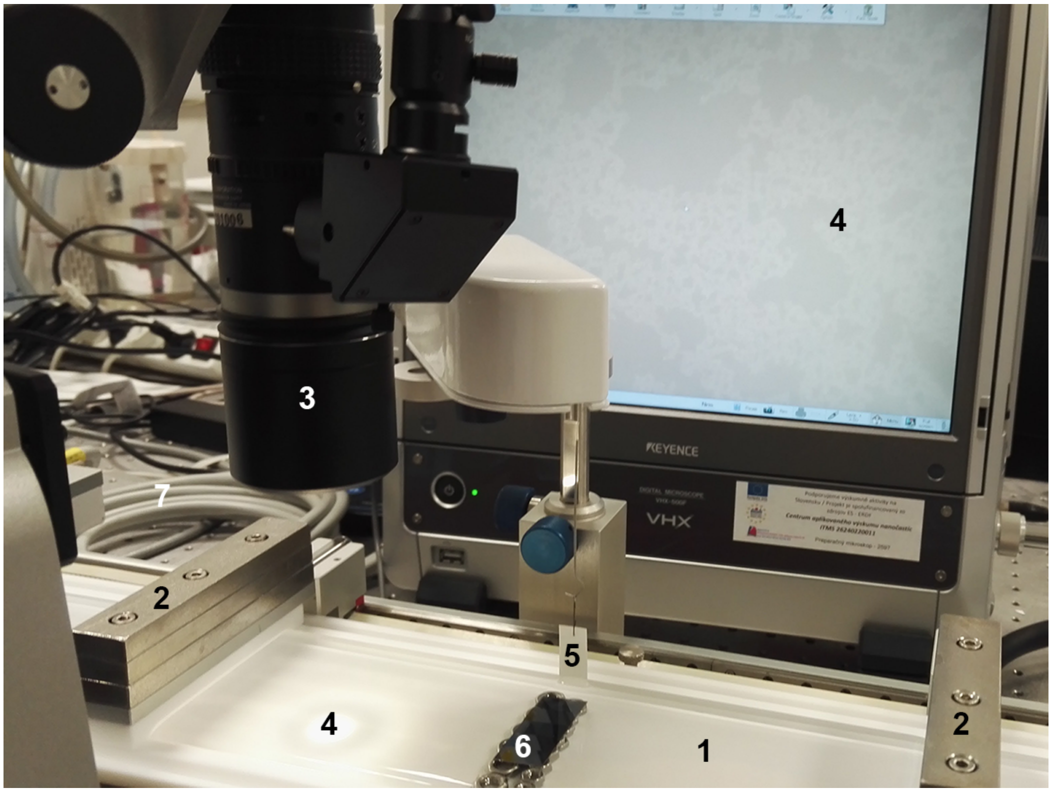

Figure S2. Image of Langmuir–Schaefer setup for FLG deposition. Numbers indicate individual components of the whole trough. (1) Trough top with injected FLG suspension, (2) movable barriers, (3) optical microscope, (4) visual output, (5) surface pressure sensor, (6) Si/SiO_2 substrates for deposition, and (7) suction pump.

*The surface pressure Π was monitored by the so-called Wilhelmy plate. It was a rectangular plate, made out of paper partially immersed in water subphase. The following Figure S3 illustrates the Wilhelmy plate.

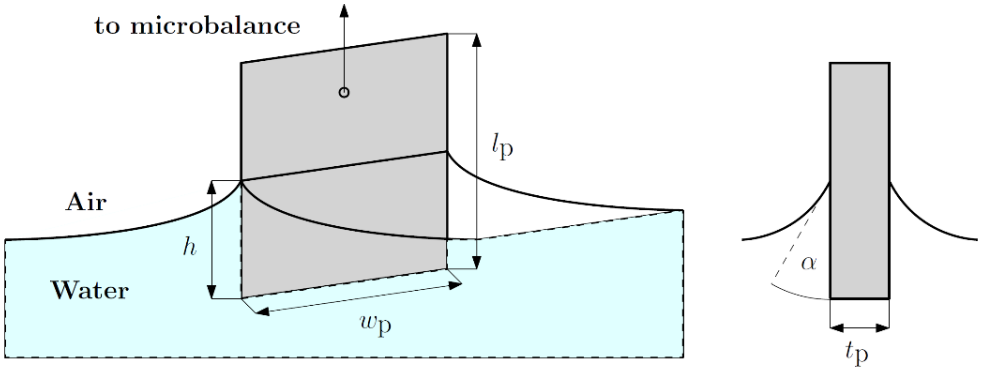

Figure S3. Wilhelmy plate scheme. The perspective view and the side view.

The force acting on this plate is sum of three force contributions; the gravity F_{G} and the surface tension F_{ST}, both are acting downwards, whereas the buoyancy F_{B} acting on the plate is acting upwards. This can be expressed by following equations:

$$F = F_{\mathrm{G}} + F_{\mathrm{ST}} - F_{\mathrm{B}} = m_{\mathrm{p}}g + \gamma \cos\alpha\, P - m_{\mathrm{l}}g,$$

for a rectangular plate of dimensions l_{p}, w_{p}, t_p, material density ρ_{p}, and perimeter P immersed to a depth h in a liquid of density ρ_{l} the net force is given by:

$$F = \rho_{\mathrm{p}} g l_{\mathrm{p}} w_{\mathrm{p}} t_{\mathrm{p}} + 2\gamma\left(t_{\mathrm{p}} + w_{\mathrm{p}}\right)\cos\alpha - \rho_l g t_{\mathrm{l}} w_{\mathrm{l}} h,$$

where γ is the surface tension of the liquid, α is the contact angle on the solid plate and g is the gravitational constant. By this approach it is possible to measure surface pressure by measuring the change of force F acting on a plate with and without a molecular film at the surface. During a deposition, Wilhelmy plate is completely wetted after a while, that means $\alpha = 0$ and cos 0 = 1. The surface pressure can be subsequently obtained from following equations:

$$F_0 = F_{\mathrm{G}} + 2\gamma_0\left(t_{\mathrm{p}} + w_{\mathrm{p}}\right) - F_{\mathrm{B}}$$

and

$$F = F_{\mathrm{G}} + 2\gamma\left(t_{\mathrm{p}} + w_{\mathrm{p}}\right) - F_{\mathrm{B}},$$

giving the final relation for surface pressure connecting the change in force and the change in surface tension

$$\Pi = \gamma_0 - \gamma = \frac{F_0 - F}{2\left(t_{\mathrm{p}} + w_{\mathrm{p}}\right)}.$$

The sensitivity is further increased by using a very thin plate, so that $t_{\mathrm{p}} \ll w_{\mathrm{p}}$, and giving the following equation

$$\Delta\gamma = \frac{\Delta F}{2w_{\mathrm{p}}}.$$

The force is then determined by measuring the changes in the mass of the plate, which is directly coupled to a sensitive microbalance.[2]

Figure S4 shows experimental setup for the second modified Langmuir–Schaefer deposition which started by injecting [Cu(dbm)$_2$] onto a subphase (also deionised water) surface (1). The movable barriers (2) were slowly closed and reduced the surface area of a trough top (1). The Wilhelmy plate made out of paper (3) was used to monitor the surface pressure. The (4) represents the graphene-covered Si/SiO$_2$ substrate from previous FLG deposition. And finally, the suction pump (5), by which the water was pumped out, and thus the water level was lowered and the deposition done. The term modified stands for the deposition carried out by elevating and tilting the substrate by a flat metal washer placed under the graphene-covered substrate.

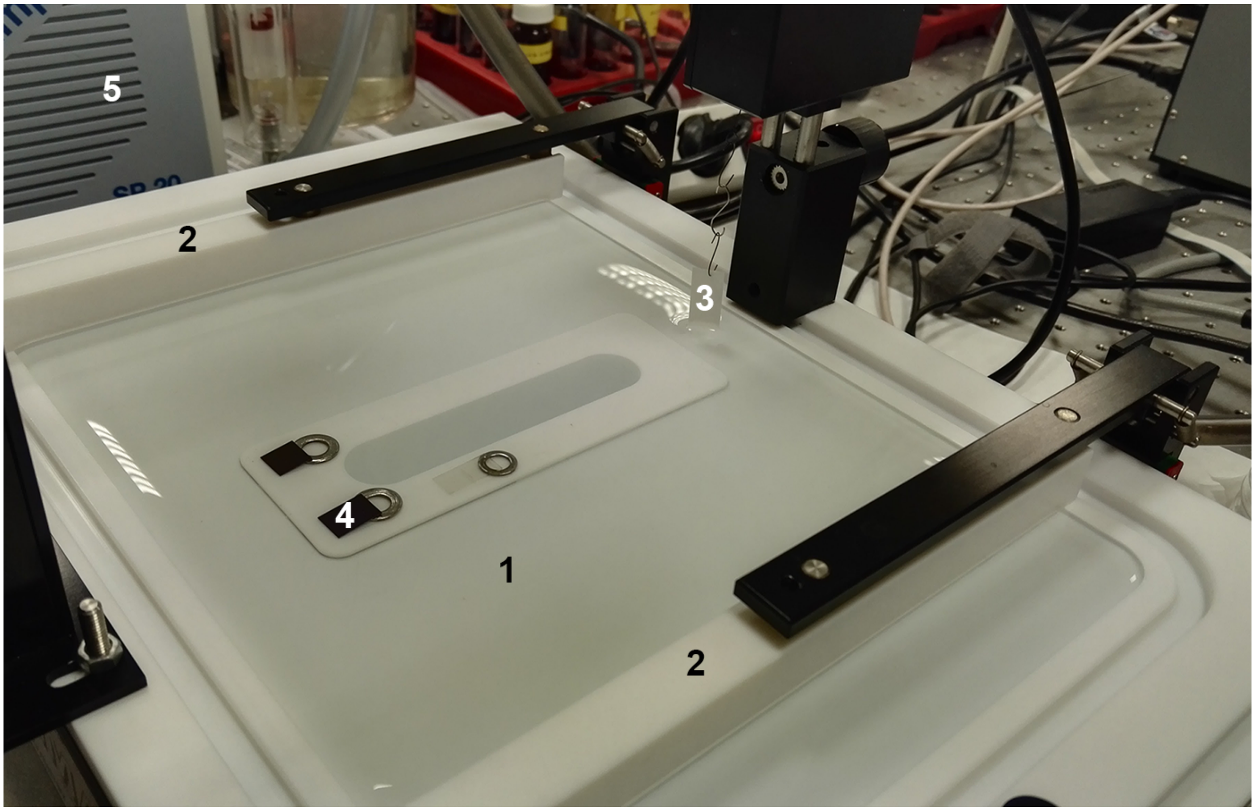

Figure S4. Image of Langmuir–Schaefer setup for molecular deposition. Numbers indicate individual components of the whole deposition trough. (1) Trough top with injected molecular complexes, (2) movable barriers, (3) surface pressure sensor, (4) graphene-covered Si/SiO$_2$ substrate, and (5) suction pump.

The second molecular deposition took place in the trough with the total area *A* of 483.3 cm^2, and the lowest speed set for closing the barriers of 20.4 cm^2/min.

By this deposition, the 5 mM solution of [Cu(dbm)$_2$] in chloroform was deposited onto FLG-covered substrate in a controlled wet-chemistry manner. This multiple modified Langmuir–Schaefer deposition is scalable, reproducible, and presents a feasible deposition route.

3) Isotherms obtained during blank deposition of [Cu(dbm)$_2$] without substrates and the actual deposition.

When the molecular layer was compressed on the water surface, it underwent phase transformations. These changes were observed by monitoring the surface pressure Π as a function of the area occupied by the film A. Figure S4 shows this diagram and is unique for every compound and gives information about the formation of the layer. It is common, in such a plot, to divide the film area A by the total number of molecules N on the water surface to obtain the area per molecule as follows:

$$a = \frac{A}{N} = \frac{A}{N_A c V},$$

where A is the actual trough top area, N_A is Avogadro constant, c is the molar concentration of the solution, and V is the volume injected onto a water subphase. The molar concentration for both 'Finding' and 'Deposition' of [Cu(dbm)$_2$] was 5 mM. The volume $V_{FINDING}$ = 750 μL and $V_{DEPOSITION}$ = 1400 μL. The isotherm was a very first characteristic of the compound regarding the deposition step. During the 'Finding' deposition, the isotherm was estimated to be at Π = 25 mN/m (i.e. where the curve starts to saturate). This pressure was then applied during the actual deposition onto substrates. The process of finding a correct isotherm is crucial for the formation of the ideal coverage. This was, however, an uneasy task for metal compounds. Surface pressure is given as follows: $\Pi = \gamma_0 - \gamma$, where γ_0 is the surface tension of the pure liquid and γ is the surface tension of the film-covered surface.

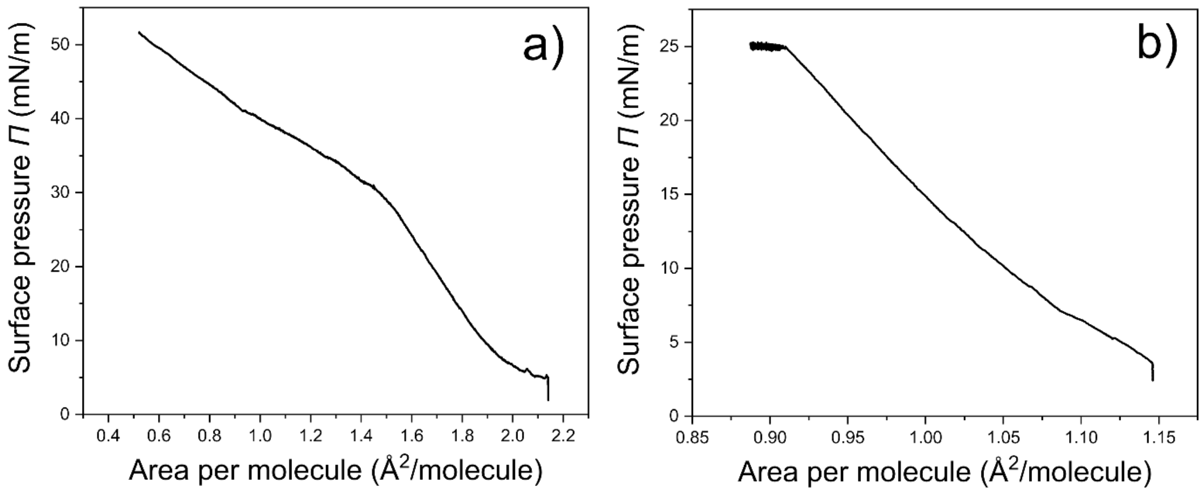

Figure S5. Diagram a) and b) show isotherms (a curve on a Π-A isotherm diagram at the constant room temperature) for [Cu(dbm)$_2$] obtained during the blank deposition and actual deposition onto substrates, respectively.

4) SEM and AFM characterisation of deposited [Cu(dbm)$_2$] on FLG.

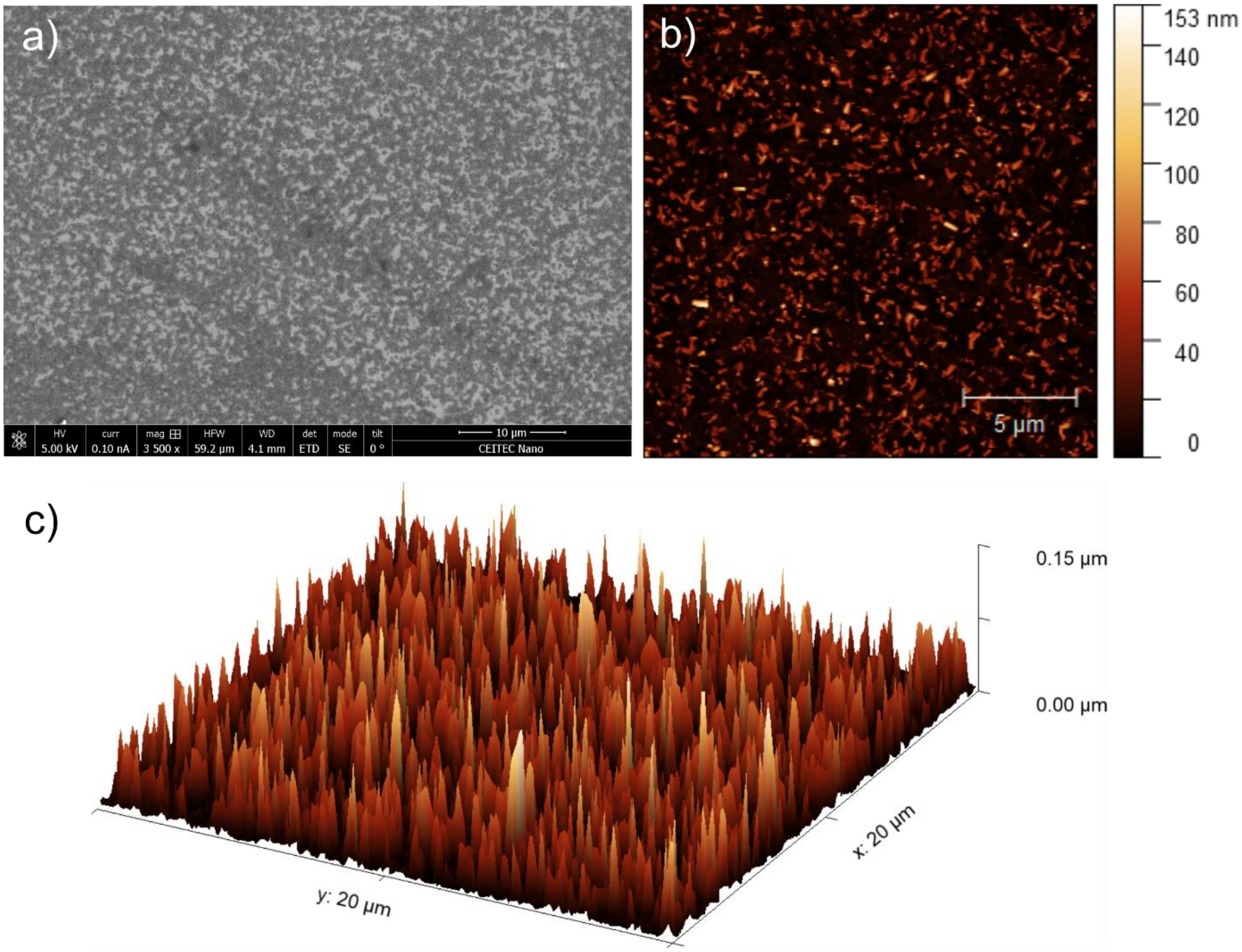

Figure S6. Characterisation of deposited [Cu(dbm)2] on FLG. a) SEM image obtained at 5 kV and equipped with secondary electrons detector, b) AFM topography image c) extracted 3D morphology image.

5) High-frequency ESR setup scheme.

Figure S7 illustrates the main setup components of HF-ESR spectrometer located at the University of Stuttgart, Germany. The tuneable microwave source (1) provided radiation of variable frequency: $\nu = 82 - 1100$ GHz, which was propagated by a quasi-optics (2). The higher frequencies were accessed by amplifying and multiplying the microwave base frequency: $\nu = 8 - 12$ GHz. The variable temperature insert (3), $T = 1.8 - 300$ K, was put in a tunable superconducting magnet (4) capable of magnetic field up to ±17 T. The microwave detection was provided by an InSb bolometer (5) cooled by the liquid nitrogen and helium in order to increase its sensitivity.

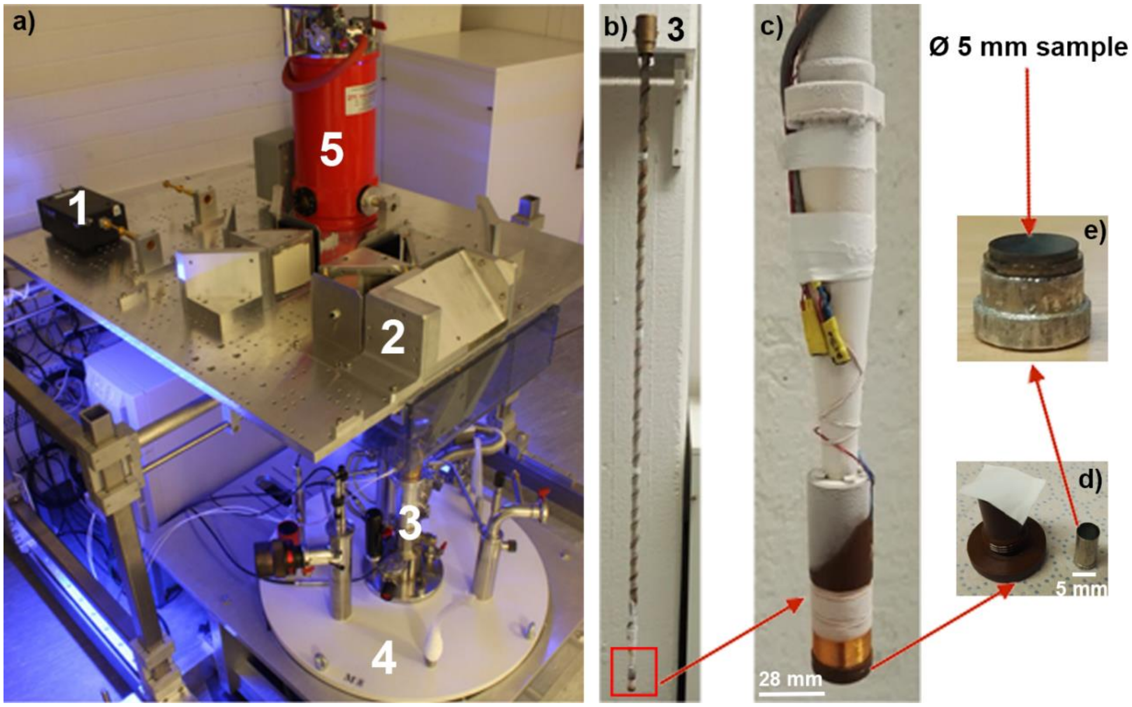

Figure S7. The HF-ESR spectrometer. a) Indication of setup components, b) variable temperature insert (VTI), c) detail of sample-rod head with modulation coil, at which the alternating current (AC) with known noise frequency was applied and formed a lock-in amplification of signal (the first derivative of absorption signal was detected), d) sample insert with support, and e) Ø5 mm sample in the form of a pressed pellet (for a bulk powder measurements) or a thin film (deposited [Cu(dbm)$_2$] on FLG-covered substrate).

5) Table S8. Raman comparison of peaks found with their assignment.

**Si/SiO$_2$ (cm^{-1})**	**FLG (cm^{-1})**	**[Cu(dbm)$_2$] powder (cm^{-1})**	**[Cu(dbm)$_2$] on FLG (cm^{-1})**	**Peak intensity**	**Reference[3] peaks (cm^{-1})**	**Peak assignment**
		3254	3251	weak		*2D*' band, νCHα
		3065	3067	medium-weak	3064	20b, 20a
	2953		2935	weak		*G* + *D* band
	2709		2711	strong		*G*' band
	1621		1622	shoulder		*D*' band
	1588	1600	1596	overlapping, very strong	1596	8a, νsC O(i.p.), G band
		1562		very weak	1554	νsC O(i.p.), 8b
		1525	1525	weak	1523	νaCCαC, δCHα, 19a
		1493	1493	strong	1492	19a
		1445	1443	medium	1443	19b,νsC=O(i.p.)
		1377		very weak	1375	19b, νaC=O
	1356		1357	very strong		*D* band
		1318	1319	very strong	1317	14, νsCCαC
		1292	1292	very strong	1290	νsCCαC, νsC-Ph, 14
		1233	1232	medium	1232	δCHα, νaC-Ph, 9a
		1187	1187	medium	1189	9a
		1168	1167	medium	1166	9a
		1155	1151	weak	1151	15
		1129	1126	weak	1126	νC-Ph, δCHα, 18a
		1071	1071	medium	1069	18b, νsCCαC(i.p.)
		1029		shoulder	1029	18a
		1026	1025	weak	1025	18a, νsCCαC(i.p.)
		1003	1003	very strong	1002	12
979			983	overlapping, weak	988	12, 2 TO phonons
943		949	948	overlapping, strong	948	δCCαC, 17a, 2 TO phonons
		923		very weak	922	17b
		845		very weak	841	10a
825			827	weak		2 LO phonons
		790	791	medium	790	Γ, 6a
		697	694	weak	693	δOCPh, 6a
669		669	669	overlapping, weak	668	4, 2 LA phonons
618		618	618	overlapping, weak	618	6b, 2 LA phonons
		566	564	weak	567	νsO-Cu-O(i.p.), 6a
521			522	very strong		TO phonon band
		462		very weak	459	16b, νaO-Cu-O
434			433	weak		LO phonons
		405	402	very weak	403	16a
		362	368	very weak	361	δO-Cu-O, δC-Ph
304			304	strong		2 TA phonons
		256	255	weak	255	νsO-Cu-O
		224	228	weak	222	δC-Ph, δO-Cu-O
		195	196	weak	197	γC-Ph, τO-Cu-O
		162	163	weak	164	νdbm-Cu-dbm
		133	131	very weak	132	τO-Cu-O
		107	107	very weak	113	δdbm-Cu-dbm

Supporting Information References

1. HRUBÝ, Jakub. Preparation and Characterization of Graphene Based Hybrid Materials. Brno, 2017.91p. Master's thesis. Brno University of Technology. Faculty of Mechanical Engineering. Supervised by Ing. Petr NEUGEBAUER, Ph.D.
2. Petty MC. Langmuir-Blodgett Films: An Introduction. Cambridge University Press; 1996. 234 p.
3. A. R. Nekoei, M. Vakili, M. Hakimi-Tabar, S. F. Tayyari, R. Afzali and H. G. Kjaergaard, Spectrochimica Acta - Part A: Molecular and Biomolecular Spectroscopy, 2014, 128, 272–279.
